# Microscopic Insight into the Structure–Processing–Property Relationships of Core–Shell Structured Dialcohol Cellulose Nanoparticles

**DOI:** 10.1021/acsabm.2c00505

**Published:** 2022-10-04

**Authors:** Aleksandar Y. Mehandzhiyski, Emile Engel, Per A. Larsson, Giada Lo Re, Igor V. Zozoulenko

**Affiliations:** † Laboratory of Organic Electronics, Department of Science and Technology, Linköping University, SE-601 74 Norrköping, Sweden; ‡ Department of Fiber and Polymer Technology, KTH Royal Institute of Technology, Teknikringen 56, SE-100 44 Stockholm, Sweden; § FibRe − Centre for Lignocellulose-Based Thermoplastics, Department of Fiber and Polymer Technology, School of Engineering Sciences in Chemistry, Biotechnology and Health, KTH Royal Institute of Technology, SE-100 44 Stockholm, Sweden; ∥ Department of Industrial and Materials Science, Chalmers University of Technology, SE-412 96 Gothenburg, Sweden; ⊥ FibRe − Centre for Lignocellulose-Based Thermoplastics, Department of Chemistry and Chemical Engineering, Chalmers University of Technology, SE-412 96 Gothenburg, Sweden; # Wallenberg Wood Science Center, Linköping University, SE-601 74 Norrköping, Sweden

**Keywords:** dialcohol cellulose, molecular dynamics, melt processing, core−shell structure, mechanical shearing

## Abstract

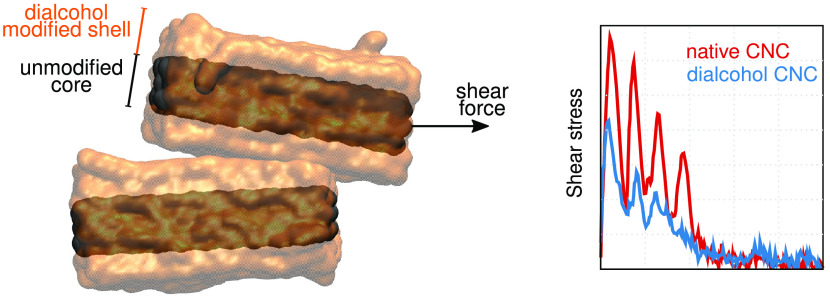

In the quest to develop sustainable and environmentally friendly materials, cellulose is a promising alternative to synthetic polymers. However, native cellulose, in contrast to many synthetic polymers, cannot be melt-processed with traditional techniques because, upon heating, it degrades before it melts. One way to improve the thermoplasticity of cellulose, in the form of cellulose fibers, is through chemical modification, for example, to dialcohol cellulose fibers. To better understand the importance of molecular interactions during melt processing of such modified fibers, we undertook a molecular dynamics study of dialcohol cellulose nanocrystals with different degrees of modification. We investigated the structure of the nanocrystals as well as their interactions with a neighboring nanocrystal during mechanical shearing, Our simulations showed that the stress, interfacial stiffness, hydrogen-bond network, and cellulose conformations during shearing are highly dependent on the degree of modification, water layers between the crystals, and temperature. The melt processing of dialcohol cellulose with different degrees of modification and/or water content in the samples was investigated experimentally by fiber extrusion with water used as a plasticizer. The melt processing was easier when increasing the degree of modification and/or water content in the samples, which was in agreement with the conclusions derived from the molecular modeling. The measured friction between the two crystals after the modification of native cellulose to dialcohol cellulose, in some cases, halved (compared to native cellulose) and is also reduced with increasing temperature. Our results demonstrate that molecular modeling of modified nanocellulose fibers can provide fundamental information on the structure–property relationships of these materials and thus is valuable for the development of new cellulose-based biomaterials.

## 1 Introduction

Modified cellulose is a potential green alternative to petroleum-derived synthetic polymers. One of the main limitations of native cellulose, when considered as an alternative to so-called “plastics”, is that native cellulose, upon heating, degrades before it melts. However, native cellulose can be chemically modified to depress the glass-transition temperature of the material and thereby impart not only thermoplasticity but in some cases also melt processability. One such chemical modification is to partially convert the cellulose in fibers to dialcohol cellulose. Such partially modified fibers (dialcohol fibers) exhibit high ductility and thermoplastic features.^1−4^ Additionally, it has been demonstrated that 100% of dialcohol fibers plasticized by water up to 90 wt % of dialcohol fiber content can be melt-processed by twin-screw extrusion and subsequent injection molding.^5^

The partial conversion of native cellulose fibers to dialcohol cellulose involves a two-step heterogeneous reaction of partial oxidation, coupled with cleavage of the C2–C3 bond on the glucose unit, followed by total reduction of the oxidized moieties.^2,6^ It is hypothesized that, under heterogeneous conditions, the cellulose nanofibril (CNF) surfaces inside the fiber wall are first to react. The reaction then proceeds from the outer part of each CNF inward, generating core–shell structured CNFs, where the shell comprises highly modified dialcohol cellulose, while the core is essentially native cellulose.^2,7^

Melt-processed parts of dialcohol fibers have been investigated experimentally using a range of techniques, including thermal analysis, mechanical testing, scanning electron microscopy, small-angle and wide-angle X-ray scattering analysis, and X-ray tomography.^5^ The investigation of structure–property relationships has subsequently expanded to include the effects of processing conditions on melt processability. By melt processability, we refer to the ease by which dialcohol fibers can be processed at elevated temperatures using typical methods such as extrusion and injection molding. However, the broad range of data collected through experimentation does not fully explain the role of intermolecular interactions during processing, and in turn how these interactions, obviously important to the melt processability of dialcohol fibers, are controlled through choice of degree of modification, water content, and temperature of processing. There are hence limitations to our understanding of the interactions between CNFs and their shorter counterparts cellulose nanocrystals (CNCs), based solely on experimental data.

Molecular dynamics modeling is a powerful tool to investigate further the relationship between cellulose interfibrillar interactions and interactions with the surrounding plasticizing water at different temperatures and degrees of modification (simulating different processing conditions) and to advance our understanding of this system. Recently, molecular dynamics (MD) simulations were applied to study the stick-slip behavior of CNCs,^8^ the effect of surface modification on the interactions between the CNCs and the resulting bundling process,^9^ and the adhesion properties of CNC on graphene oxide.^10^ These studies provide valuable understanding of the microscopic mechanisms of friction and adhesion in cellulose systems. However, to the best of our knowledge, MD simulations of dialcohol-modified CNCs and their properties have not been performed yet. Therefore, here we present the first example of a molecular modeling approach applied to dialcohol cellulose. Based on previous experimental studies,^2,7^ we design a computational core–shell model for dialcohol cellulose by varying the surface accessible moieties on a cellulose nanocrystal level according to the proposed reaction mechanism of progressively commuting C2–C3 cellulose bonds to dialcohol *via* oxidation reduction.^1,2^ Using atomistic molecular dynamics simulations, we investigate dialcohol cellulose CNC–CNC and CNC–water intermolecular interactions and the interactions between adjacent dialcohol-modified cellulose nanocrystals (DA-CNCs) as model particles. We also experimentally investigate the melt processing of dialcohol cellulose with different degrees of modification and/or water content using the fiber extrusion technique. The experimental data are analyzed based on the simulated results for the intermolecular interaction and CNC conformations, and a good agreement is found. Our results demonstrate that molecular modeling of modified nanocellulose fibers can provide fundamental information on the structure–property relationships of these materials and thus is valuable for the development of new cellulose-based biomaterials.

## 2 Models and Methods

### 2.1 Molecular Dynamics Simulations

#### 2.1.1 Dialcohol Cellulose Model

**Figure 1:**
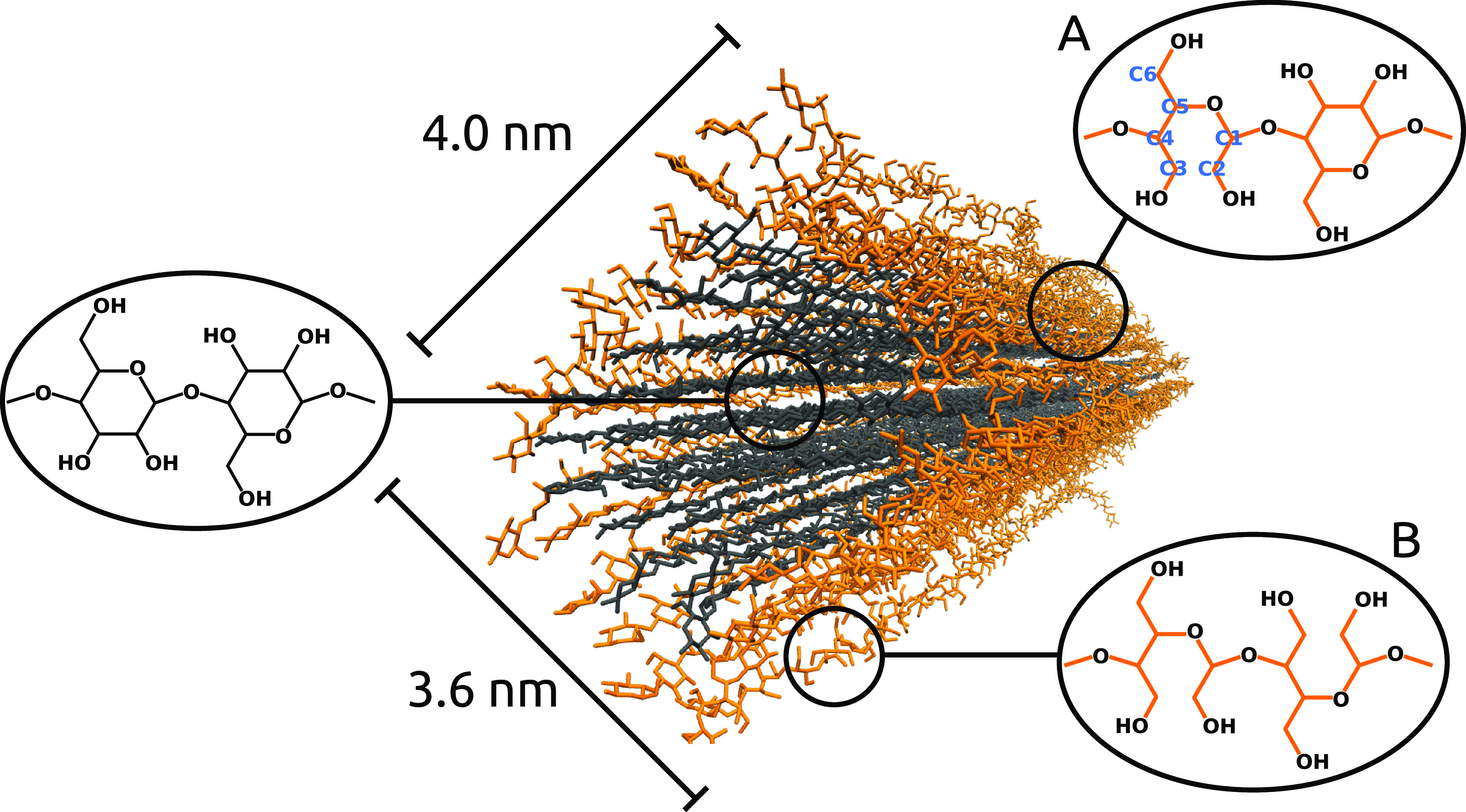
Dialcohol CNC with a degree of modification of 25%. Chemical structures of cellulose chains in the core region (gray) and dialcohol chains located at the shell (outer layer) of the crystal (orange). Two different models are proposed for the modification of the C2–C3 bonds in dialcohol cellulose: alternating dialcohol-modified surface as illustrated in (A) and fully dialcohol-modified surface (B).

Experimentally, we have modified and processed fibers, which comprise modified CNFs (modified inside the fiber, *via* a heterogeneous reaction). We processed partially modified diacohol cellulose fibers, but these are made up of tightly bound CNFs. Therefore, we used a model of native and dialcohol-modified cellulose nanocrystals (CNCs and DA-CNCs, respectively) as representative of very short CNFs. CNCs and DA-CNCs were built in the Iβ cellulose allomorph where 49 cellulose chains are placed in a square cross section, *i.e.*, 7 × 7 cellulose chains, thus resulting in a CNC with dimensions of 3.6 × 4.0 nm^2^. It is noteworthy that the true nature of cellulose crystallites is still a subject of debate. Our CNC model is based on NMR and SAXS model pioneered by Larsson et al.^11,12^ A chain length of 40 glucose units was used, resulting in a CNC length of 20.8 nm. This chain length was chosen to keep a reasonable computational time while at the same time to explore a large enough system. The degree of modification (DoM) for DA-CNCs was set to 0% (*i.e.*, a native CNC), 25, 40, and 100%. DA-CNCs are presumed to have a core–shell structure where dialcohol chains form a shell around an unmodified core.^1,2,13^ The MD DA-CNC model with a DoM of 25% is shown in Figure 1, where the unmodified core chains are depicted in gray and the modified shell chains are shown in orange. It is not clear experimentally and indeed an ongoing challenge to determine how the modified repeating units are distributed. Thus, as a first study, for the modification, we have considered two different idealized models for dialcohol-modified chains. For the chain model (A), every second C2–C3 bond is modified, whereas in model (B), all C2–C3 bonds are modified (see Figures 1 and S1). We built these two models based on the assumption that at the beginning of the reaction only every second C2–C3 bond (case A) of the side chains is exposed to water, while the other C2–C3 bonds point toward the interior of the CNC; thus, some monomers at the surface of the CNC are modified and some are not. For example, for DoM = 25%, there are 22 exterior chains (located at the sides of the CNC, as shown in Figures 1 and S1) where every second C2–C3 bond is exposed to water, while the other C2–C3 bonds point toward the interior of the CNC. Thus, the latter are not accessible for oxidation at the beginning of the reaction. In addition, at the top and bottom of the CNC, there are two chains where all of their C2–C3 bonds are pointing outward into the water and making them accessible for oxidation (case B), see Figure S1. Therefore, for the case DoM = 25%, the CNC consists of 22 side chains where every second C2–C3 bond is modified (case A) and two chains (top and bottom) where all bonds are modified (case B). By applying the same procedure also to the second layer, we obtained 40% modification, and finally by removing all C2–C3 bonds, we have a 100% modified CNC, as shown in Figure S1. We used a combination of case A and case B for the 25 and 40% DoM. It should be noted that any DoM less than 100% can be obtained by different combinations of modifying the glucose C2–C3 bonds. For example, 100% of the C2–C3 bonds in the outermost layer can be broken, or 80% of the outermost layer and only 20% of the chains in the second layer. Thus, different combinations can be imagined achieving the same DoM.

The unmodified or modified CNCs were placed centrally in a simulation box with dimensions of 20 × 20 × 40 nm^3^ and water was then added to the system. An initial equilibration and rescaling of the simulation box were carried out in the constant number of particles, pressure, and temperature (NPT) ensemble for 1 ns, which was followed by 5 ns constant number of particles, volume, and temperature (NVT) equilibration. The potential energy during the 5 ns equilibration has reached a plateau, and it is shown in Figure S2 in the Supporting Information. The final run where all of the statistics were collected for the analysis of the results was performed in the NPT (constant number of particles, pressure, and temperature) ensemble for 100 ns. The temperature in the simulations was set to 25 °C.

#### 2.1.2 Steered MD Simulations

The force needed to shear one CNC over another can be obtained by steered MD simulations.^14^ The shear force is then divided by the contact area between the two CNCs to obtain the stress of shearing. It should be noted that we used a constant value for the contact area during the sliding of the CNCs; thus, the stress presented here is not the true stress but the engineering stress. The contact area of the unmodified fiber was calculated by the solvent-accessible surface area (SASA) which in our systems is 65.5 nm^2^. In the steered MD simulations, the length of each CNC was 10.4 nm (20 glucose units) to keep reasonable computational time. Initially, the two crystals are placed next to each other with a surface-to-surface distance of 3 or 8 Å to accommodate one or three layers of water molecules between the surfaces, respectively. After that, the system was solvated with water, energy minimization was performed, and then, NVT and NPT equilibrations were performed, having the position of one of the chains in the two CNCs restrained, to equilibrate the water molecules around the CNCs. A final NPT equilibration was then performed for 5 ns where instead the positions of the nine innermost chains were restrained. Thus, the two outer layers, which are involved in the interaction between the CNCs, were left to freely equilibrate. In the steered MD simulations, one CNC was restrained (inner 9 chains) and the other CNC was pulled at a rate of 1 nm ns^–1^ and a spring constant of 10 000 kJ mol^–1^ nm^–2^.

Two different cases of shearing the two CNCs were explored: pulling in the axial direction (Ax) and in the transverse (Tr) directions of the CNC. In addition, two different temperatures were explored, 25 and 100 °C, for the case of separation of 3 Å separation (at 25 °C a separation of 8 Å was also used). Thus, several systems were explored and were abbreviated as follows: Ax_3@25; Ax_3@100; Ax_8@25; Tr_3@25; Tr_3@100; Tr_8@25. The two-letter code indicates the direction of pulling (axial and transverse): 3 and 8 indicate the surface-to-surface distance, and the last numbers indicate the temperature in the simulations.

#### 2.1.3 Simulation Details

The MD simulations were performed with the GROMACS simulation package version 2021.^15,16^ The OPLS-AA force field for carbohydrates was used to describe all atomic species.^17,18^ The TIP3P^19^ water model was used to describe water in the simulations. All bonds involving hydrogen atoms were constrained by the LINCS algorithm^20^ and the simulation time step was set to 2 fs. The GROMACS tools for hydrogen bonds and radial distribution function, RDF (gmx hbond and gmx rdf), were used to calculate the number of hydrogen bonds in the simulations and the RDF. To calculate the structure factors, which are closely related to X-ray diffraction patterns, we first calculated the RDFs between the atom pairs C–O, C–H, and O–H. In these calculations, we excluded atom pairs belonging to the same chain; thus, the obtained RDFs are between the cellulose chains of the CNC. Then, the structure factors were calculated from the Fourier transform of these RDFs.^21,22^ The smooth particle-mesh Ewald summations (PME)^23^ algorithm was used for electrostatics with a cutoff distance of 1.2 nm; the van der Waals cutoff was also set to 1.2 nm. The velocity rescaling algorithm^24^ was used to control the temperature with a coupling constant of 100 fs, and the pressure was controlled by the Berendsen barostat^25^ with a coupling constant of 2 ps. The simulation snapshots were prepared with VMD.^26^

### 2.2 Materials

Bleached softwood kraft fibers (K48) were supplied by SCA Forest Products (Östrand pulp mill, Timrå, Sweden). The pulp was mechanically beaten in a Voith mill to an energy input of 160 Wh kg^–1^. Fines, *i.e.*, small-particle material generated during beating (5–10% by mass), were removed from the beaten fibers by filtration through a 200 mesh metal screen, using a Britt Dynamic Drainage Jar (Paper Research Materials, Seattle). Sodium metaperiodate (supplied by Alfa Aesar), sodium borohydride, and hydroxylamine hydrochloride (both supplied by Sigma-Aldrich) were of 98% purity. Other chemicals, such as hydrochloric acid, sodium hydroxide, sodium phosphate, isopropanol, and ethanol were supplied by Sigma-Aldrich and were of reagent grade.

### 2.3 Preparation of DAC Fibers

#### 2.3.1 Chemical Modification of Fibers

Beaten cellulose fibers were partially modified to dialcohol cellulose. To achieve this, the fibers were first partially oxidized to dialdehyde cellulose using sodium metaperiodate, followed by total reduction of aldehydes to hydroxyls by treatment with sodium borohydride, in a similar manner to earlier protocols.^2^^,^^16^ For the oxidation reaction, fibers were suspended in 6.3% (v/v) isopropanol in water at a fiber concentration of 15 g L^–1^. Isopropanol served as a radical scavenger to limit unwanted side reactions.^17^ The fibers in suspension were then partially oxidized with 1.35 g of sodium metaperiodate per gram of fibers, under constant stirring, in one instance for 28 h and in another instance for 38 h. The oxidation reaction was stopped by washing the fibers with deionized water until the filtrate conductivity was 10 μS cm^–1^ or lower. The fibers were then resuspended to 15 g L^–1^ in 0.1 M sodium phosphate monobasic (to prevent the pH from increasing beyond pH 10, limiting alkaline depolymerization upon addition of sodium borohydride) and treated with 0.5 g of sodium borohydride per gram of fibers, under constant stirring, for 2 h. The reduction reaction was stopped by washing for the oxidation reaction.

#### 2.3.2 Determination of the Degree of Modification

The degree of modification was estimated by determining the aldehyde content after periodate oxidation. Aldehyde content was determined in triplicate by a well-established protocol based on reaction with hydroxylamine hydrochloride.^3,27^ Since all aldehydes are finally reduced, the degree of modification after periodate oxidation is taken as the final degree of modification to dialcohol cellulose.

### 2.4 Melt Processing

#### 2.4.1 Conditioning of DAC Fibers

DAC fibers were suspended in water, cast onto Teflon film, and dried at 50 °C in a forced air oven, resulting in translucent films (e.g., Figure S3). Strips of approximately 10 mm in width were cut from these films and were conditioned for 7 days under a controlled relative humidity of either 30% or more than 90%. Relative humidity of 30% was achieved by conditioning the material in a sealed vessel, which contained an open saturated solution of magnesium chloride. Relative humidity exceeding 90% was achieved using a sealed vessel containing deionized water.

#### 2.4.2 Melt Processing of DAC Fibers by Twin-Screw Extrusion

Melt processing was done at 100 °C using a DSM Xplore Micro 5cc twin-screw mini-extruder (Heerlen, Netherlands). The instrument is designed for small-scale continuous melt-processing experiments. It comprises conical screws within a vertical 5 cm^3^ barrel and allows for recirculation of the material before extrusion *via* a 2 mm die. Recirculation and extrusion are controlled *via* a manual valve. Conditioned strips were fed into the l extruder at a screw speed of 30 rpm. After feeding the extruder, the screw speed was increased to 100 rpm and the material was recirculated until the axial force stabilized before the die was opened. The axial force of the screws, proportional to the viscosity at the processing conditions, was recorded throughout the extrusion experiment.

## 3 Results

### 3.1 Molecular Modeling of Core–Shell Structured Dialcohol Cellulose CNC

**Figure 2:**
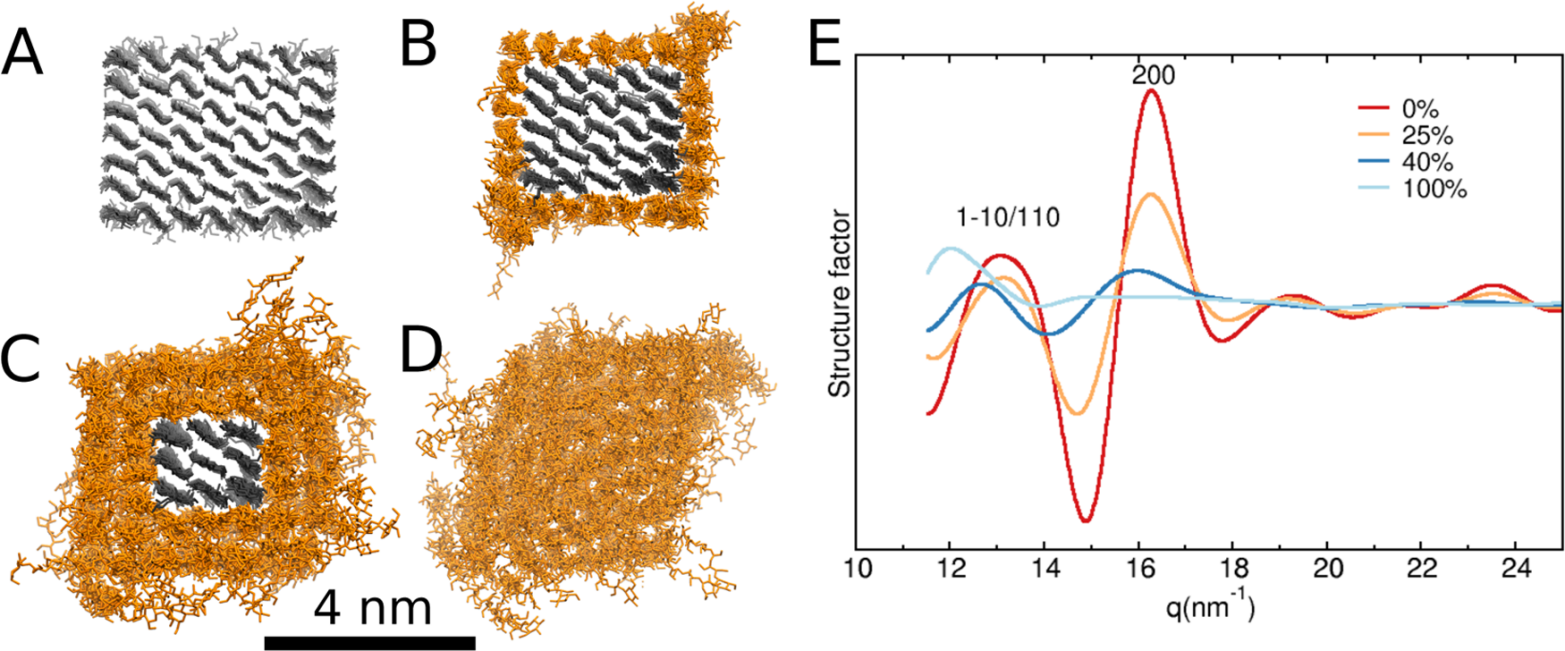
CNC cross sections at different degrees of modification: (A) 0%, (B) 25%, (C) 40%, and (D) 100%. (E) Structure factor of the studied systems. Gray color depicts unmodified cellulose chains, and orange color depicts dialcohol-modified chains.

In this section, we first take a closer look at the structure of a single-dialcohol CNC. Figure 2A–D shows the cross section of the model CNC for different levels of modification, from 0 to 100%. The simulations suggest that the unmodified chains (gray) retain their highly crystalline structure, while the dialcohol cellulose chains (orange) at the surface are significantly more disordered. It can also be seen that, at a DoM of 100%, the shape of the cross section changes from rectangular to rhomboid shape. Figure 2E shows the structure factors calculated for the different levels of modification. For the unmodified CNC (0% DoM), two main peaks are easily distinguishable, (200) at 16.2 nm^–1^ and (110/1–10) at 13.0 nm^–1^. These peaks are characteristic of cellulose Iβ and are in good agreement with experimental X-ray diffraction results.^28,29^ (It is noteworthy that (110) and (/1–10) peaks are merged in a single peak. This is consistent with previous calculations^30^ where these peaks become discernible for CNCs with the dimensions 8 × 8 chains). With the increase of DoM, the (200) peak decreases and completely disappears at a DoM of 100%, while the (110/1–10) peak decreases and shifts to lower *q*-values (reciprocal distance units—1/nm). This behavior is expected due to the increase in dialcohol cellulose content and hence the decrease in the number of unmodified chains at the core of the crystal. The same trend has also been observed experimentally for dialcohol fibers, which indicates that our molecular model is consistent with experimental findings.^2^

**Figure 3:**
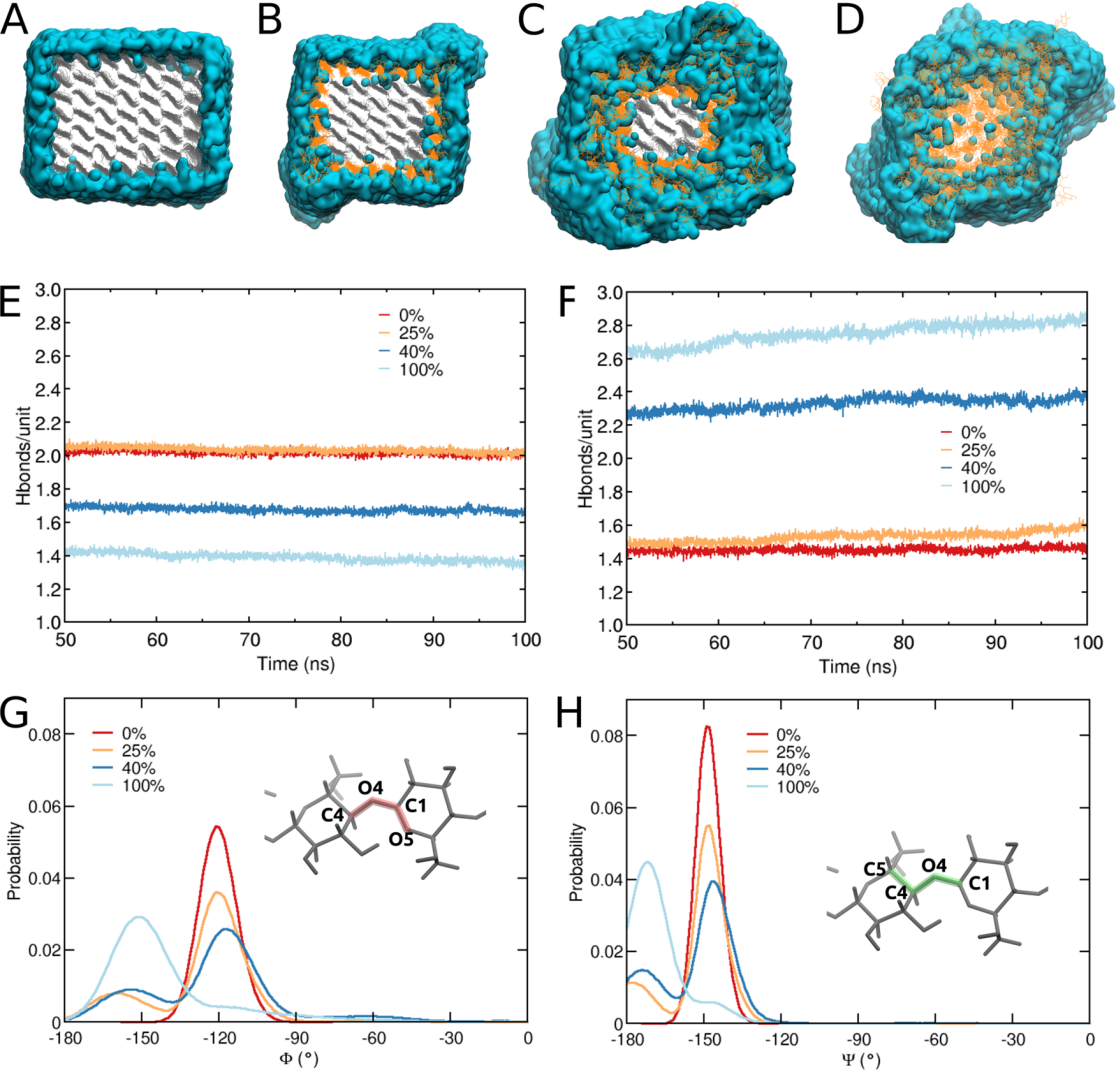
Distribution of water molecules around CNCs (A–D) for degree of modifications of (A) 0%, (B) 25%, (C) 40%, and (D) 100%. The water molecules within 6 Å of cellulose are only shown. Gray color depicts the unmodified cellulose chains and orange color the dialcohol-modified chains; water is depicted in blue. Number of hydrogen bonds per glucose unit as a function of simulation time, for (E) within the CNC (cellulose/cellulose chains) and (F) between a CNC and surrounding water molecules (CNC/water); probability distributions of the torsion angles (G) Φ and (H) Ψ, describing the conformation around the glucosidic bonds.

Under the hypothesis that surface interactions play a key role in the melt processability of the dialcohol cellulose fibers, we assessed in detail the effect of degree of modification on the water content and the hydrogen bonding within the CNCs and between individual CNCs and surrounding water. Figure 3A–C shows the water distribution around the CNC for different DoMs, where only the water molecules (visualized in blue) within 6 Å of the cellulose chains are shown. Water molecules cannot penetrate inside the dense unmodified CNC (DoM = 0%), while with the increase in DoM, a significant amount of water penetrates inside the disordered dialcohol cellulose shell. However, the water molecules still cannot penetrate inside the core of unmodified cellulose for DoM of 25 and 40% and are hence only located in the modified regions. For a DoM of 100%, water molecules are also able to reach the middle of the crystal.

Figure 3E–F shows the number of hydrogen bonds per glucose unit within the CNC and the number of bonds between the CNC and water molecules, respectively. The number of hydrogen bonds (glucose-to-glucose) within the crystal (Figure 3E) is around two for a DoM of 0% and remains stable throughout the simulation. The increase in DoM leads to a significant decrease of this number, and, moreover, it decreases steadily throughout the simulation. The decrease in the number of hydrogen bonds correlates with a concomitant increase in the number of hydrogen bonds between the CNC and water (Figure 3F). At a DoM of 0%, the number of hydrogen bonds is constant throughout the simulation, while an increasing DoM facilitates penetration of water molecules into the CNC and therefore the number of hydrogen bonds with water steadily increases throughout the simulation. For DoM over 25%, a constant value for the number of bonds was not reached during the simulation. Therefore, 40 and 100% DoM are expected to have even more water adsorbed in the CNC upon longer simulation times. For a DoM of 100%, it is possible that the CNC will be completely dissolved if given enough time. The hypothesis of full solubility is also supported by experimental observations.^31^ However, we decided that the simulation time of 100 ns was enough to determine the core–shell structure of the DA-CNC. Therefore, we believe that the structure presented here is a fair representation of experimentally derived DA-CNC and that we can use these model CNCs to study the interactions between modified CNC.

To describe the conformation of the cellulose chains around the glucosidic linkages, we have calculated the torsion angles Φ (C4-O4-C1-O5) and Ψ (C5-C4-O4-C1)^32^ for all DoMs and presented them in Figure 3G,H, respectively. For DoM of 0%, both angles Φ and Ψ have a single distribution located at −120 and −150°, respectively. With the increase of DoM, in both cases, we observe widening of the peak and appearance of a second peak at 150° (Φ) and 170° (Ψ). The torsion angle Φ has a nonzero probability in the range −180 to 0° for DoM of 40 and 100%. This suggests that the oxidation of the C2–C3 bond significantly enhance the flexibility of the chain and the rotation around the glucosidic bond is much easier. In addition, the appearance of a second peak in the distribution suggests a change in the conformation of the dialcohol-modified chains.

### 3.2 Experimental Melt Processing of DAC Fibers

Chemical modification as described in Section 2.3 yielded DAC fibers of 31 and 41 ± 1% degrees of modification (after 28 and 38 h reaction time, respectively). It bears mentioning that some variability arises from the process of washing fibers to stop reactions.^2,33^ Conditioning the 41% DoM fibers at 30% relative humidity (RH) resulted in a moisture content of 5.3 ± 0.2%, while the fibers conditioned at more than 90% relative humidity had a moisture content of 35.1 ± 0.4%.

**Figure 4:**
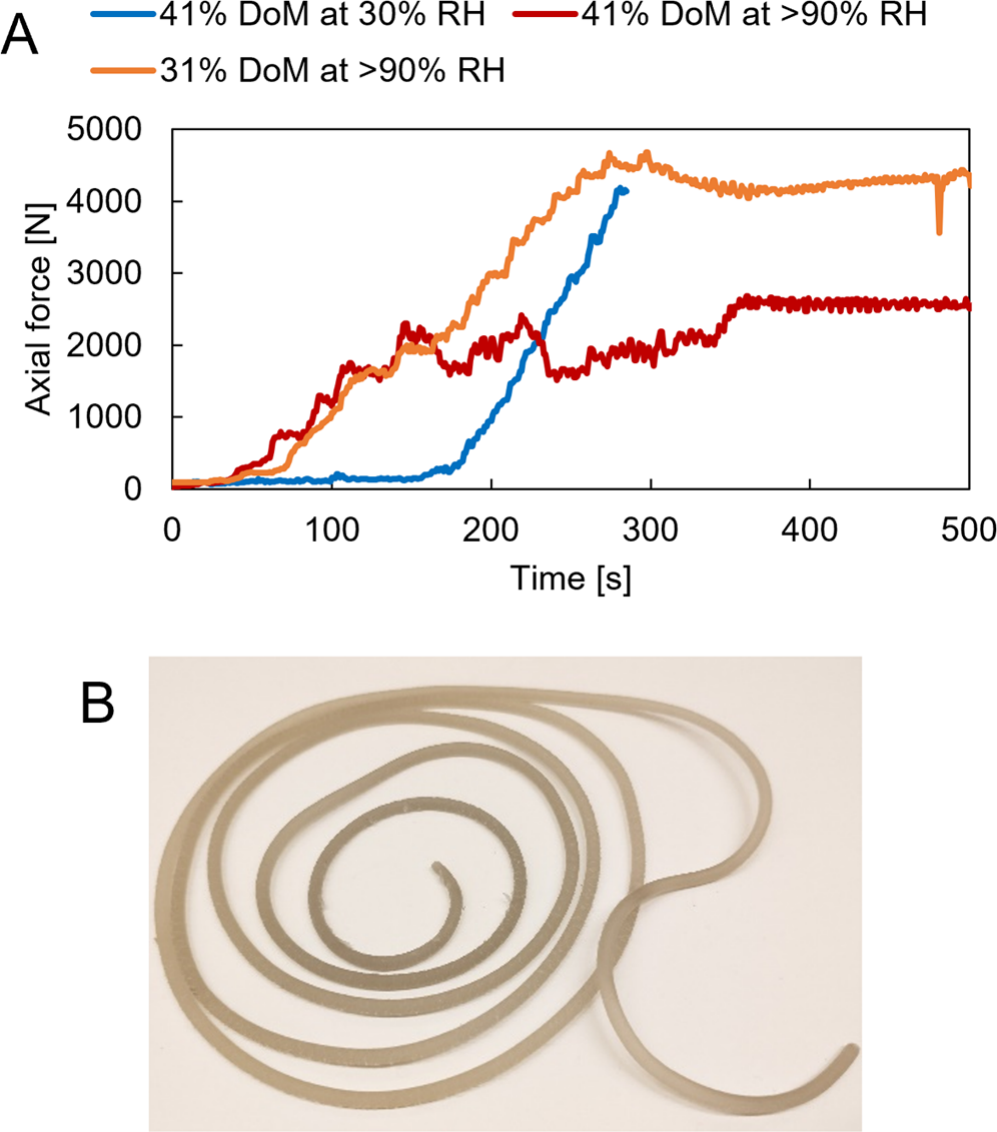
Axial force as a function of time for the feeding of the extruder and the melt processing of DAC fibers of 31 or 41% DoM. The 31% DoM fibers had been conditioned at above 90% RH, while the 41% DoM fibers were conditioned at either 30% or above 90% RH. (A) Full data range for the three samples investigated. (B) Extruded filament obtained from melt processing of DAC fibers that had been conditioned at a relative humidity greater than 90%.

Data plotted as axial force as a function of processing time in the mini-extruder, *i.e.*, obtained from melt processing of DAC fibers (note: macroscopic fibers and not CNCs), are provided in Figure 4A. Note that the instrument is limited to 5000 N of axial force. By melt processing we refer to processing by typical procedures, such as melt extrusion, compression molding, and injection molding, where materials are softened by heating for the purpose of shaping. Here, we have processed by extrusion. We describe the softening of DAC fibers by heating. No claims are made regarding true melting of DAC fibers. Failure of the extrusion instrument occurs when the 5000 N limit is reached or too rapidly approached such that it is anticipated to exceed 5000 N. The 41% DoM fibers conditioned at a relative humidity of more than 90% were successfully melt processed, resulting in the material shown in Figure 4B. During the recirculation step of melt processing (Figure 4A, red curves), the axial force stabilized at approximately 2.5 kN. The moisture content of 35% achieved by this high-humidity conditioning provided adequate plasticization such that the viscosity of the material at 100 °C is low enough to facilitate melt processing with relative ease. To investigate the effect of moisture content, 41% DoM fibers were also conditioned at 30% relative humidity instead before processing. At this lower moisture content (5%), a higher axial force was generated (Figure 4A, blue curve). The axial force on the screws increased too rapidly during feeding, causing instrument failure when the maximum current was exceeded, thus activating its safety stop mechanisms not to damage the equipment from too high axial forces. Unsurprisingly, the moisture content of 5% achieved by conditioning at 30% relative humidity does not provide sufficient plasticization (such that the viscosity remains too high) to facilitate melt processing by this method. fibers at 31% DoM were conditioned at more than 90% relative humidity before processing to probe the effect of DoM. These fibers generated higher axial forces (approximately 4000–4700 N) than in the case of the 41% DoM fibers. However, the axial force did not increase too steeply, and melt processing was possible within the limits of the instrument. These experimental observations from melt processing are consistent with previous evidence for DAC fibers in paper form, where higher DoM and higher moisture content were associated with greater softening upon heating (or softening at lower temperatures) and increased ductility.^1−3^

### 3.3 Modeling of the Interactions between Two CNCs

**Figure 5:**
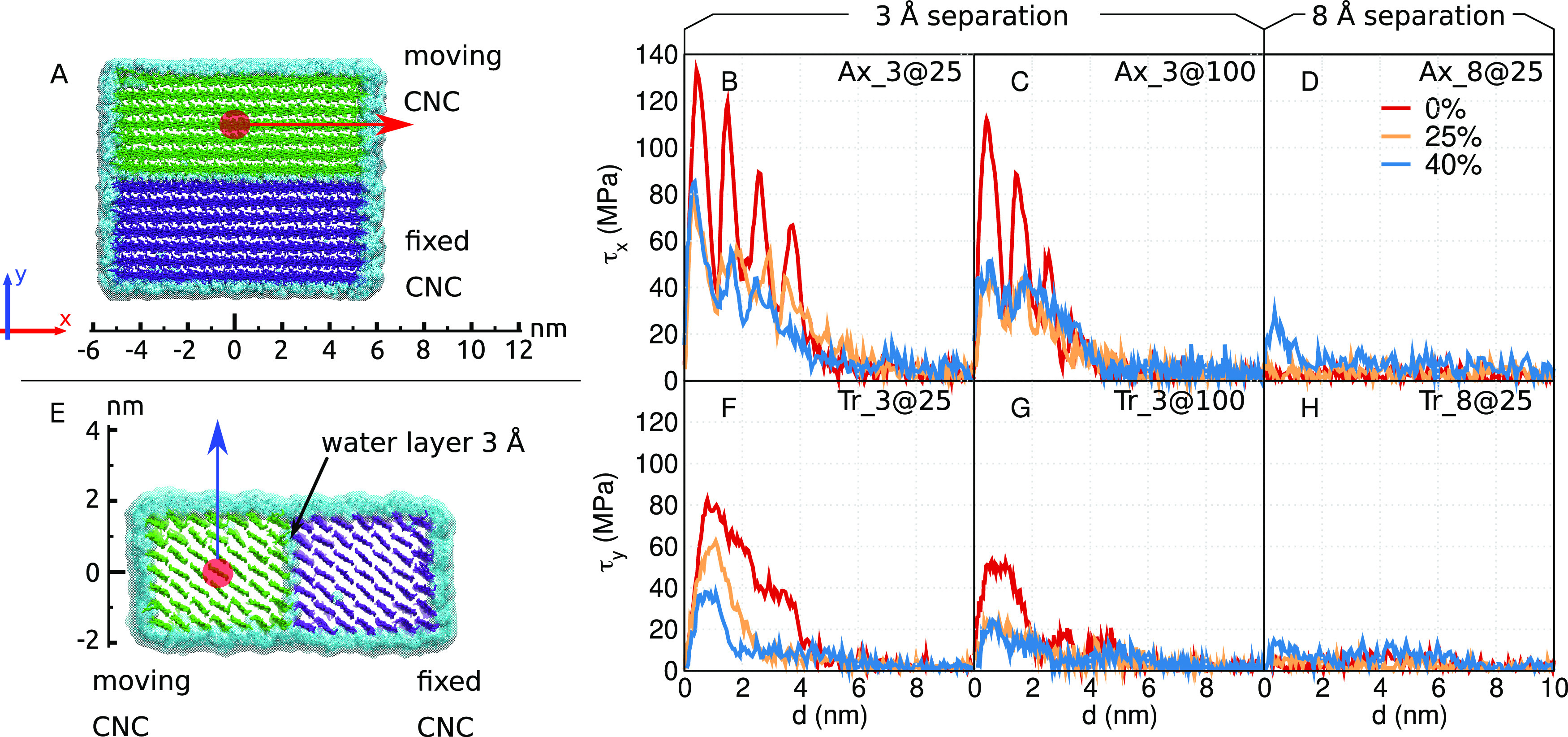
Schematic representation of the (A) axial (Ax) and (E) transverse (Tr) shear simulations, and stress–displacement curves for CNCs of different degrees of modification and system conditions (water layer and temperature) (B) Ax_3@25; (C) Ax_3@100; (D) Ax_8@25; (F) Tr_3@25; (G) Tr_3@100; (H) Tr_8@25.

Undoubtedly, chemical modification changes the way the polymer chains interact under a shear force. This is evident from the force needed to melt-process-modified cellulose fibers shown in Figure 4. To understand the molecular interactions behind this behavior, we now instead look closely at the microscopic level, using MD simulations and the DA-CNC model that we developed (Figures 2 and 3) to better understand our macroscopic system. This was done by steered MD simulations where the shear behavior of two CNCs in intimate contact with each other was investigated. Figure 5A,E schematically shows how we sheared two DA-CNFs, in the axial (Ax) or transverse (Tr) directions, respectively, and Figure 5B–D,F–H presents the stress–displacement curves of these configurations.

**Figure 6:**
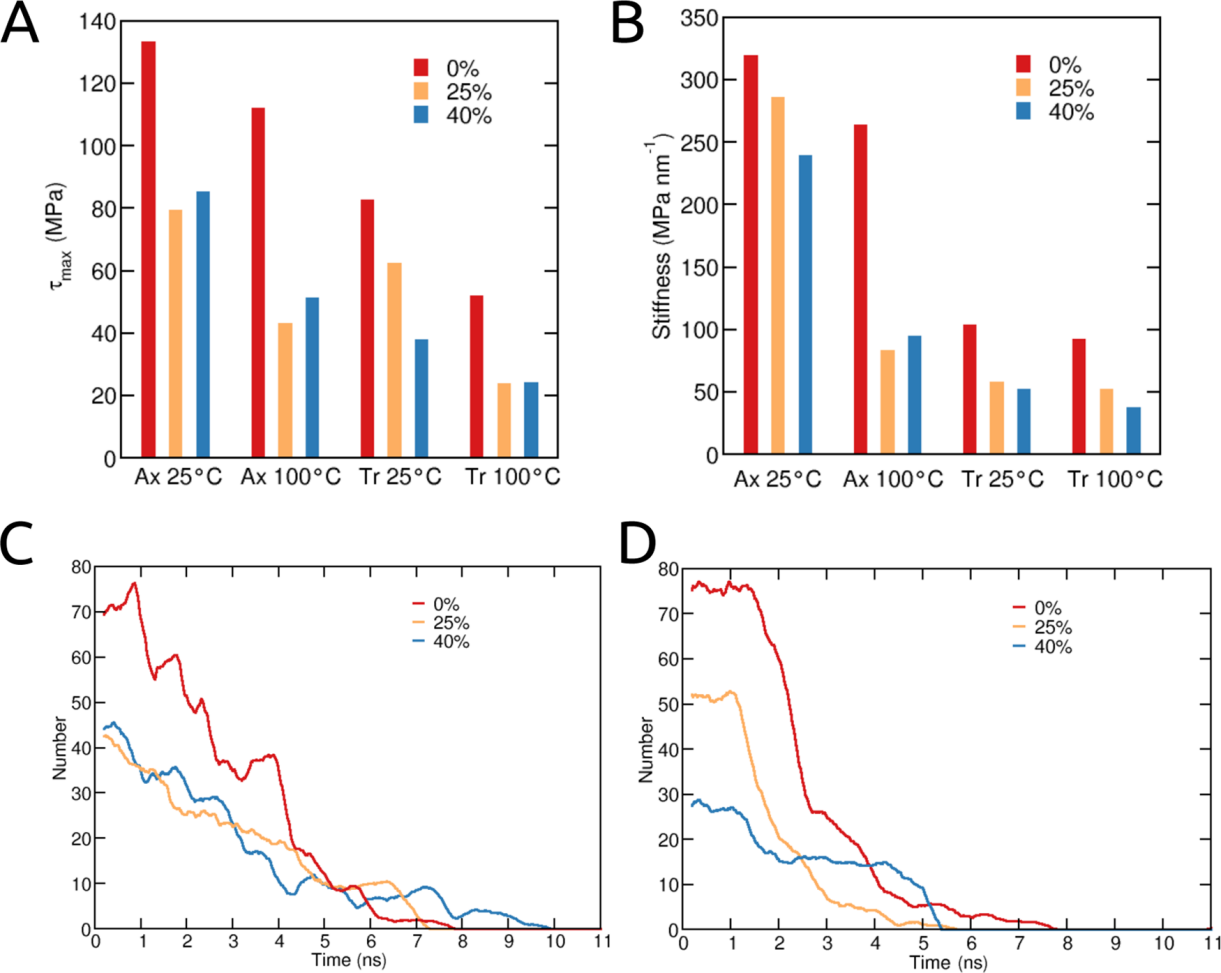
(A) Maximum shear stress (τ_max_) and (B) interfacial stiffness for the different simulation cases; different degrees of modification with a water separating layer of 3 Å. Number of hydrogen bonds between CNCs during shearing at 25 °C in (C) axial and (D) transverse directions.

Initially, a stick-slip behavior can be observed when the two CNCs are sheared past each other in the axial direction (Figure 5B,C). This stick-slip was more prominent for unmodified CNCs, in particular at lower temperature and with fewer water molecules separating the CNCs (Ax_3@25). We observed four peaks corresponding to four stick-slip events. An increased DoM to 25 and 40% not only made the stick-slip pattern less prominent; it also lowered the maximum force of shearing (τ_max_) between the two CNCs (Figure 6A). For unmodified CNCs with little water present (system Ax_3@25 and DoM = 0%), it should be noted that the maximum stress (τ_max_) decreased with every stick-slip event because (i) the finite size of the CNCs resulted in a gradually decreasing contact area between the CNCs and (ii) the moving CNC started to tilt during the pulling and the two CNCs were not kept parallel to each other throughout the simulation, further decreasing the area in contact. Increasing the temperature to 100 °C (Figures 5C and 6A) further decreases τ_max_, where the effect of temperature was more prominent for DA-CNCs than for unmodified CNCs. τ_max_ almost halved in the case of DA-CNC at high temperatures, while only a 16% decrease was observed for unmodified CNCs with increasing temperature. When the distance between the two CNCs was increased to 8 Å (Figure 5D), *i.e.*, introducing more water molecules in between the CNCs, the force needed to separate the CNCs was close to zero, that is, the two CNCs hardly feel the presence of each other. Thus, we might conclude that on the macroscopic scale the resistance to flow is induced by the network strength and fiber contacts (entanglements). A small increase in τ*_x_* (with a small peak of about 30 MPa) can be seen for small distances for system Ax_8@25 at a DoM of 40%. This is probably because the DA-CNCs interface shell region has swelled due to its increased interaction with water (Figure 3), and consequently, the distance between the two interfaces was less than 8 Å. Finally, it is worth noting that a stick-slip behavior of native CNCs was also observed by another research group, and by them studied also in further detail.^8^ In that work, the authors used a smaller and periodic CNC to investigate the stick-slip phenomena, contrary to the finite-size CNCs used in our study. Although the simulation setup in our study is somewhat different, our observed τ_max_ for native CNCs is found to be in good agreement with their findings.

The force needed to shear two CNCs in the transverse direction is lower compared to the axial shearing, as seen in Figures 5F–H and 6 A. At the lower temperature (25 °C), a trend of decreasing τ_max_ with increasing DoM can be observed in Figures 5F and 6A. An increased temperature to 100 °C makes the effect of DoM for the cases of 25 and 40% DoM practically insignificant. These results suggest that water as a plasticizer has a large effect on reducing the shear forces, so leading to lower viscosity (axial force in the experimental). However, the force needed for shearing the CNCs apart at higher temperatures is also lower for all simulated cases, regardless of DoM. By increasing the distance between the two CNCs to 8 Å, as observed for axial shearing, the two CNCs can be slid past each other without hardly applying any force at all.

Owing to the breakage of the C2–C3 bond, dialcohol cellulose chains are more flexible compared to native cellulose. Therefore, the surface of DA-CNCs is easier to deform and can more easily adapt to the applied force, which results in lower shear stress (τ_max_) upon separation of two CNCs. The interfacial stiffness (Δτ/Δ*d*) calculated from the stress–displacement curves is plotted in Figure 6B, and the stiffness of the interface of DA-CNCs is significantly lower than that of native CNCs. Moreover, temperature has a large effect on stiffness, especially for the case of axial shearing. The difference between a DoM of 25 and 40% is negligible in most cases, except for the system with less water at low temperatures (Ax_3@25). We deduce that the outermost surface layer is mostly responsible for the interactions between the two CNCs. It should also be noted that we carried out additional simulations for a DoM of 40%, where we had modified every C2–C3 bond (case B in Figure 1B) in the surface chains (the second layer stays unmodified). We then recalculated again the stress–displacement curves for the axial (Figure S4) and transverse (Figure S5) pulling directions at 25 °C, and the results suggest that it does not matter exactly how the modified chains are distributed (modifying 1 or 2 surface layers) since the calculated stress profiles are very similar in magnitude and behavior.

It was shown that hydrogen bonds play an essential role in the stick-slip behavior of two CNCs, in close proximity, that are sheared apart.^8^ Therefore, we calculated the total number of hydrogen bonds between the two model CNCs during the steered MD simulations, and the results are presented in Figure 6C,D for axial and transverse shearing, respectively. The change in hydrogen bonding observed upon increasing DoM correlates well with the change in stress–displacement behavior. An increasing DoM lowers the number of hydrogen bonds between individual CNCs because some of these bonds are now replaced by hydrogen bonds with water. This would imply, however, that the total number of hydrogen bonds in the system remains the same (Figure S6). Nevertheless, the shearing stress for larger DoM would be, to some extent, mediated by the water molecules, which could be related to the plasticizing effect^34^ of water observed experimentally for these systems.^5^ Therefore, it is suggested that hydrogen bonding is an important factor during the shearing of the two CNCs and their number is largely affected by the degree of modification.

## 4 Discussion

This section aims to connect the findings on molecular level achieved by MD simulations with macroscopic observations during melt processing. First, we examine the effect of water on the processing of DA-CNC. It was recently calculated by MD simulations that the distance between TEMPO-modified cellulose at relative humidities 30 and 80% is 5 and 8 Å, respectively.^35,36^ Thus, the relative humidity of 30% in the experiments would correspond roughly to one water layer surrounding the CNC, and it can be compared with the simulation results with a separation distance of 3 Å.^37^ In contrast, the experimental results achieved after conditioning at a relative humidity of >90%, where the increased amount of water is expected to lead to multiple water layers between the CNFs and thus separate them more effectively, will be compared with the simulations having a CNC separation distance of 8 Å. It was also previously estimated that three layers of water on the cellulose surface correspond to ca. 1 nm.^37^ The simulations clearly showed (Figure 5B–D,F–H) that for a separation distance of 8 Å, practically no force is required to shear the two CNCs past each other; thus, one CNC does not feel the presence of the other CNC. While separated by only one water layer (3 Å), a significant friction between them can be observed in the simulations. Experimentally, it was not possible to extrude DAC fibers at low moisture content, which means that there is still significant friction between the CNFs constituting the fibers, and not enough water to properly plasticize them. Therefore, we can conclude that more than one layer of water enveloping each CNC, *i.e.*, a higher water content than 5%, is necessary to provide the low friction required to extrude the material. We should also like to mention that although we completely neglect the effect of particle (fiber–fiber, CNC–CNC) entanglement in the simulations, we believe that the simulation and experimental results support each other well.

The second important factor for eased processing of DAC fibers is the degree of modification, as clearly shown in Figure 4. When processing by extrusion, higher axial force is required to convey the lower DoM material. We only observed a clear trend of decreasing shear stress with increasing DoM for system Tr_3@25, *i.e.*, less water and low temperature, where the stress at a DoM of 40% was around 30% lower than at a DoM of 25%. The calculated surface stiffness (Figure 6 B) was only slightly lower for a DoM of 40% than a DoM of 25%. However, for DA-CNCs compared to native CNCs, there was a significant decrease in shear stress and stiffness, due to the more flexible dialcohol chains in the former. To further investigate the difference between 25% and 40% DoM, we integrated the force–displacement curve to calculate the interaction energies between the CNCs, and the results are presented in Figure S7, for the different cases at 25 °C. It should be noted that this is not a rigorous method for obtaining interaction energies from molecular simulations since an enhanced sampling method such as umbrella sampling is needed to quantitatively calculate them correctly. Nevertheless, qualitatively, we could expect to observe the same trends just by integrating the force–displacement curves. Figure S7 shows that the interaction energies between DA-CNC with a DoM of 40% are lower than those between DA-CNCs with a DoM of 25%, which partially explains why it is easier to melt-process DA-CNFs with higher DoM of 41% compared with DoM of 31%.

## 5 Conclusions

In the present study, we investigated the computed structure, interactions with water, and shearing behavior of dialcohol cellulose CNCs, and proposed a relation with the experimental melt processing of the dialcohol fibers. The core–shell structure of dialcohol fibers was first simulated with molecular dynamics, and their structure factor was calculated for different degrees of CNC modification. The structure factor shows a similar trend of decreasing the crystallinity with increasing degree of modification comparable with previous X-ray scattering experiments. Thereafter, we examined the interactions of the DA-CNCs with water and showed the swelling and water penetration in the amorphous dialcohol cellulose surface region. The dialcohol cellulose surface region is expected to be amorphous based on experimental evidence that partial conversion to dialcohol cellulose reduces crystallinity. Although it is possible that 100% dialcohol cellulose could crystallize, no experimental or computational evidence exists to support the assumption of crystallization for the purpose of the present study, nor are any large domains of 100% dialcohol cellulose present. Simulated shearing of two CNCs at different conditions (temperature, degree of modification, and CNC separation) provided molecular insight into the possible mechanism by which these materials are melt-processable. The hydrogen-bond network was largely affected by the degree of modification compared to native cellulose, and it is one of the possible explanations for lower friction when the degree of modification is increased. Another factor that impacts the shearing behavior is the interfacial stiffness of the CNCs—higher degree of modification lowers the interfacial stiffness due to the larger flexibility of dialcohol cellulose chains compared to unmodified ones. In addition, highly modified fibers adsorb more water, which also affects the interfacial stiffness. The overall results from our simulations agree well with experimental melt processing where we clearly observed that dialcohol fibers are more easily extruded at high degree of modification (41%) and greater water content (relative humidity >90%).

## Supporting Information Available

Simulation setups of the CNCs with different degrees of modification and equilibration criteria; example of a film obtained from dialcohol fibers; additional shearing simulation results; total number of hydrogen bonds, and interaction energy between CNCs (PDF)
