# Supplementary material for: Microscopic Insight into the Structure–Processing–Property Relationships of Core–Shell Structured Dialcohol Cellulose Nanoparticles
Source: ACS Appl Bio Mater. 2022 Oct 4;5(10):4793–802. doi: 10.1021/acsabm.2c00505 (PMC9580023; doi:10.1021/acsabm.2c00505)
Supplement: Supplementary file 1 — mt2c00505_si_001.pdf [file mt2c00505_si_001.pdf]

Supporting Information:
Microscopic Insight into the Structure-Processing-Property Relationships of Core-Shell Structured Dialcohol Cellulose Nanoparticles

Aleksandar Y. Mehandzhiyski[1], Emile Engel[2,3], Per A. Larsson[2,3], Giada Lo Re[4,5], and Igor V. Zozoulenko[1,6*]

[1] Laboratory of Organic Electronics, Department of Science and Technology, Linköping University, SE-601 74, Norrköping, Sweden
[2] Department of Fiber and Polymer Technology, KTH Royal Institute of Technology, Teknikringen 56, SE-100 44 Stockholm, Sweden
[3] FibRe – Centre for Lignocellulose-based Thermoplastics, Department of Fibre and Polymer Technology, School of Engineering Sciences in Chemistry, Biotechnology and Health, KTH Royal Institute of Technology, SE-100 44 Stockholm, Sweden
[4] Department of Industrial and Materials Science, Chalmers University of Technology, SE-412 96, Gothenburg, Sweden
[5] FibRe – Centre for Lignocellulose-based Thermoplastics, Department of Chemistry and Chemical Engineering, Chalmers University of Technology, SE-412 96 Gothenburg, Sweden
[6]Wallenberg Wood Science Center, Linköping University, SE-601 74, Norrköping, Sweden

** corresponding author email: igor.zozoulenko@liu.se*

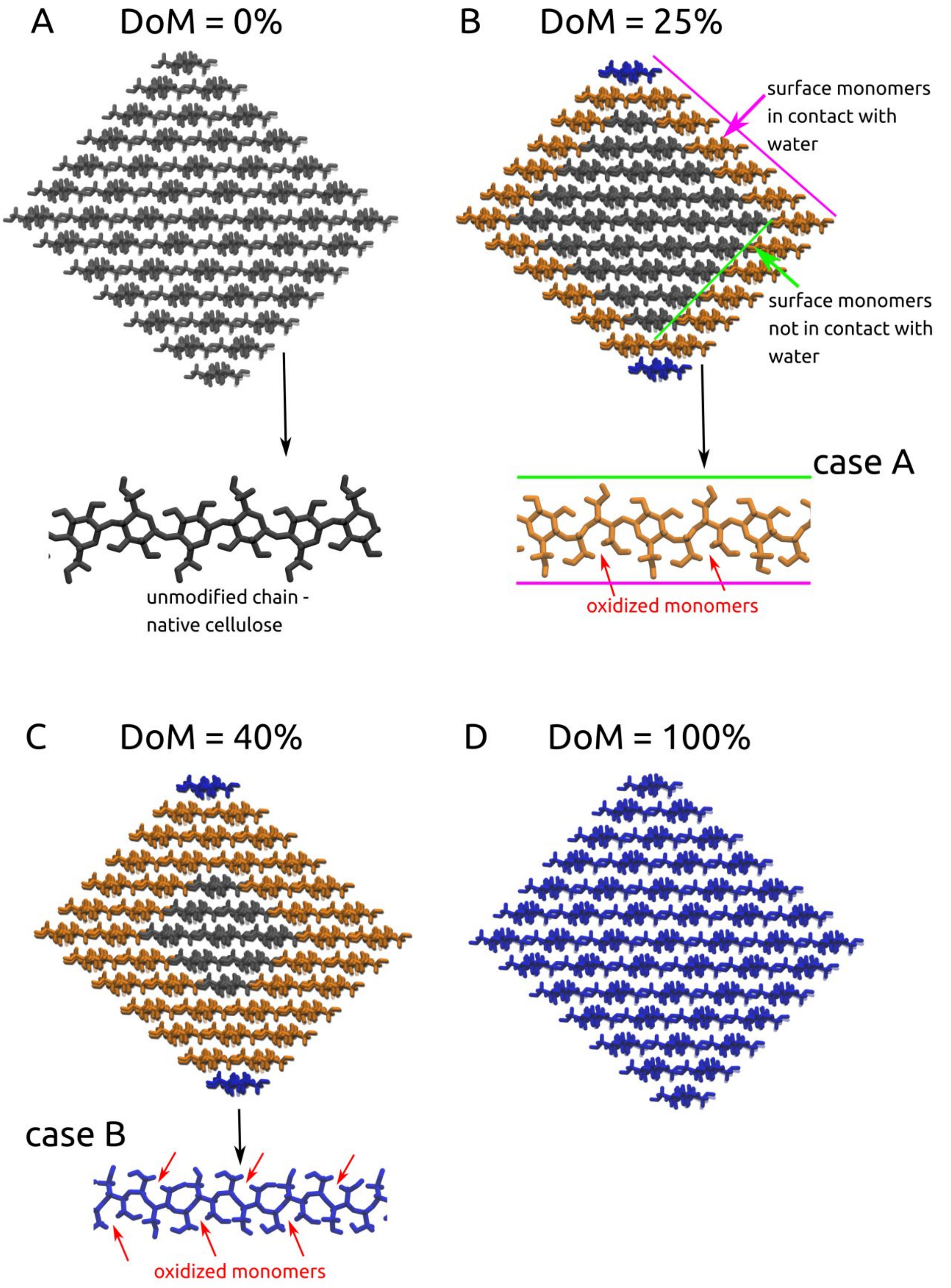

Figure S1 Visualization of the CNC models with different degree of modification (a) DoM=0%, (b) DoM=25%, (c) DoM=40% and (d) DoM=100%. Insets illustrate the models for dialcohol-modified chains: dialcohol chains with every second monomer oxidized (case A, depicted in orange) and dialcohol chains with every monomer oxidized (case B, depicted in blue). Unmodified cellulose chains are depicted in grey.

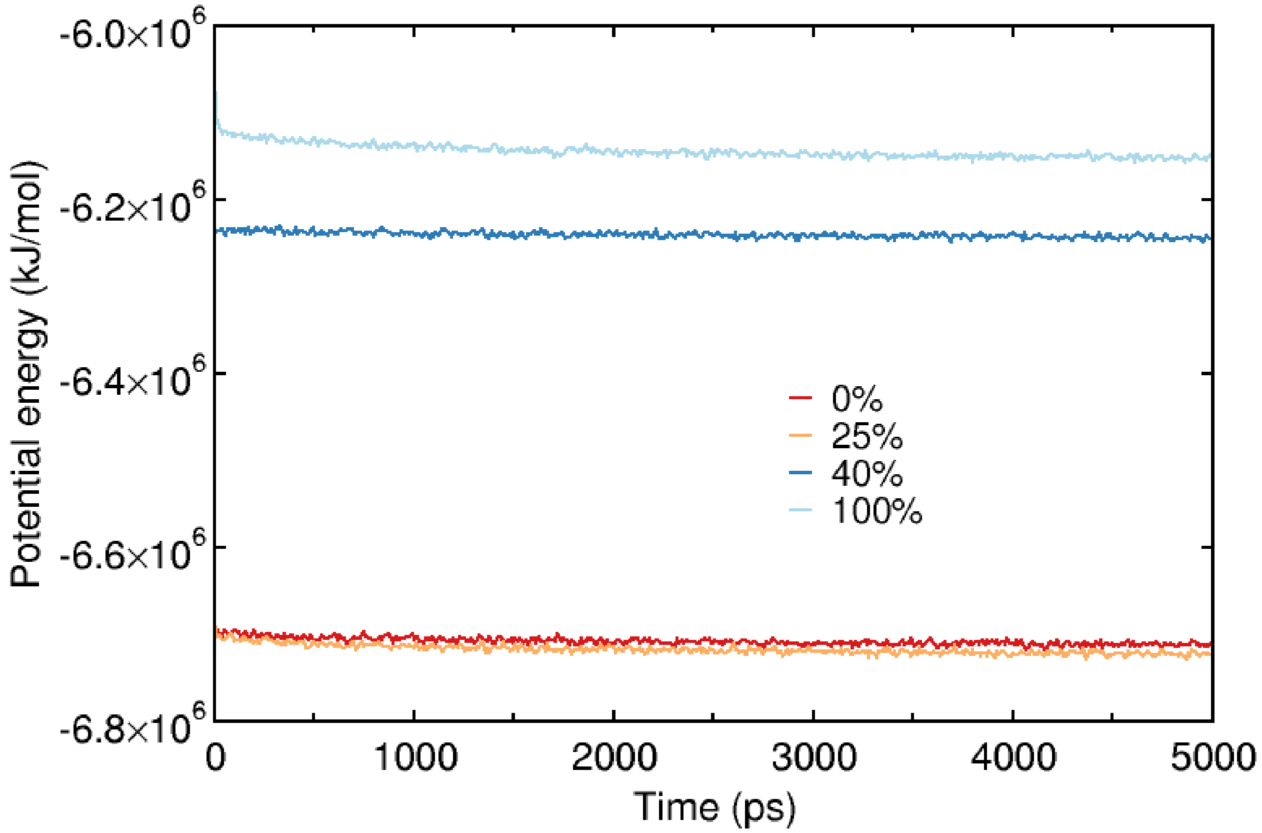

Figure S2 Potential energy during the NVT equilibration

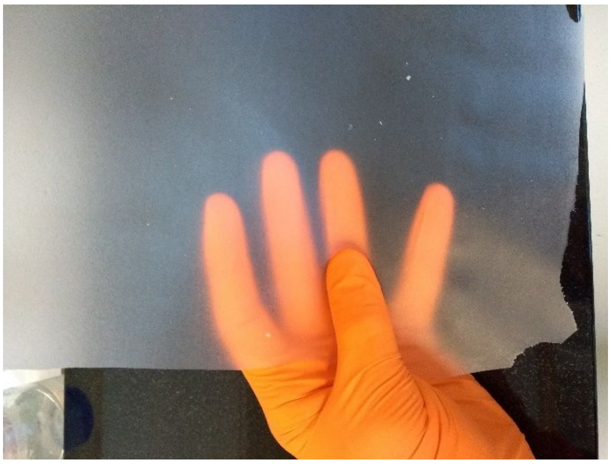

Figure S3 Example of a film obtained by casting a suspension of DAC fibres in water.

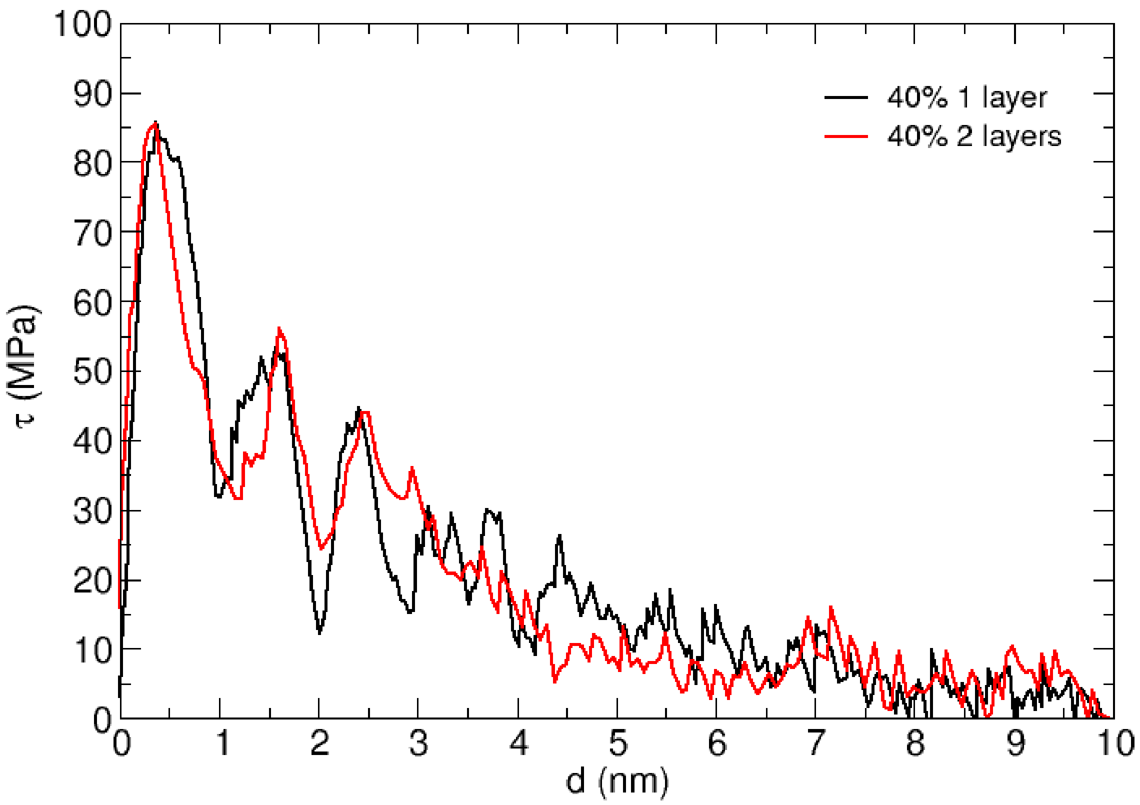

Figure S4 Comparison of axial pulling of 40% DAC when only the first layer (black) is fully modified with the case where the first and second layers are modified to 40% (red).

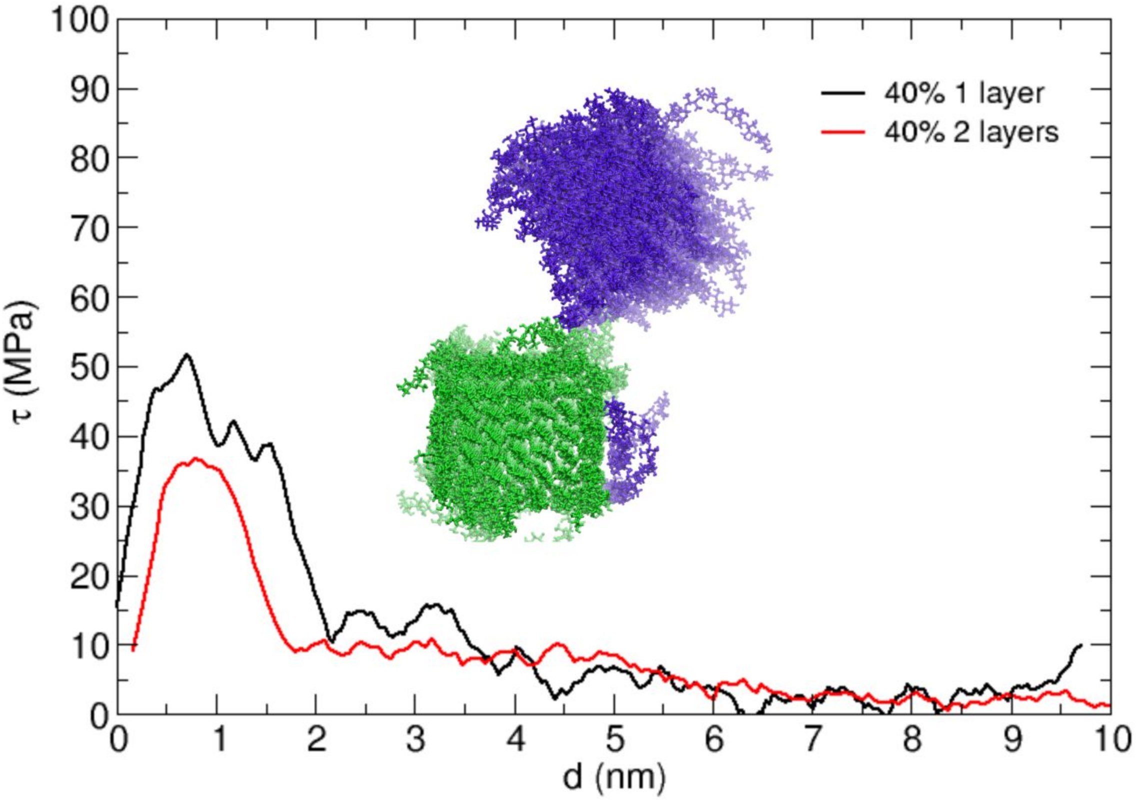

Figure S5 Comparison of perpendicular pulling of 40% DAC when only the first layer (black) is fully modified with the case where the first and second layers are modified to 50% (red). The inset simulation snapshot shows the two DA-CNC in different colors.

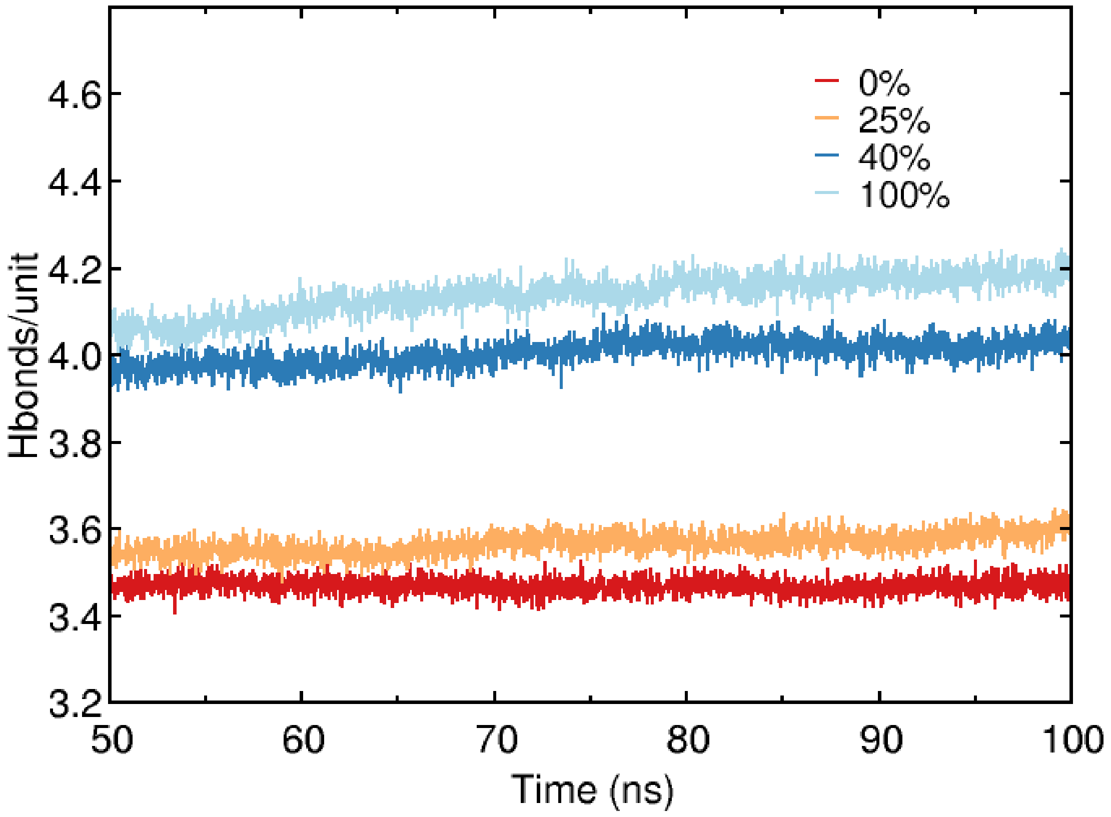

Figure S6 Total number of hydrogen bonds per glucose unit as function of simulation time

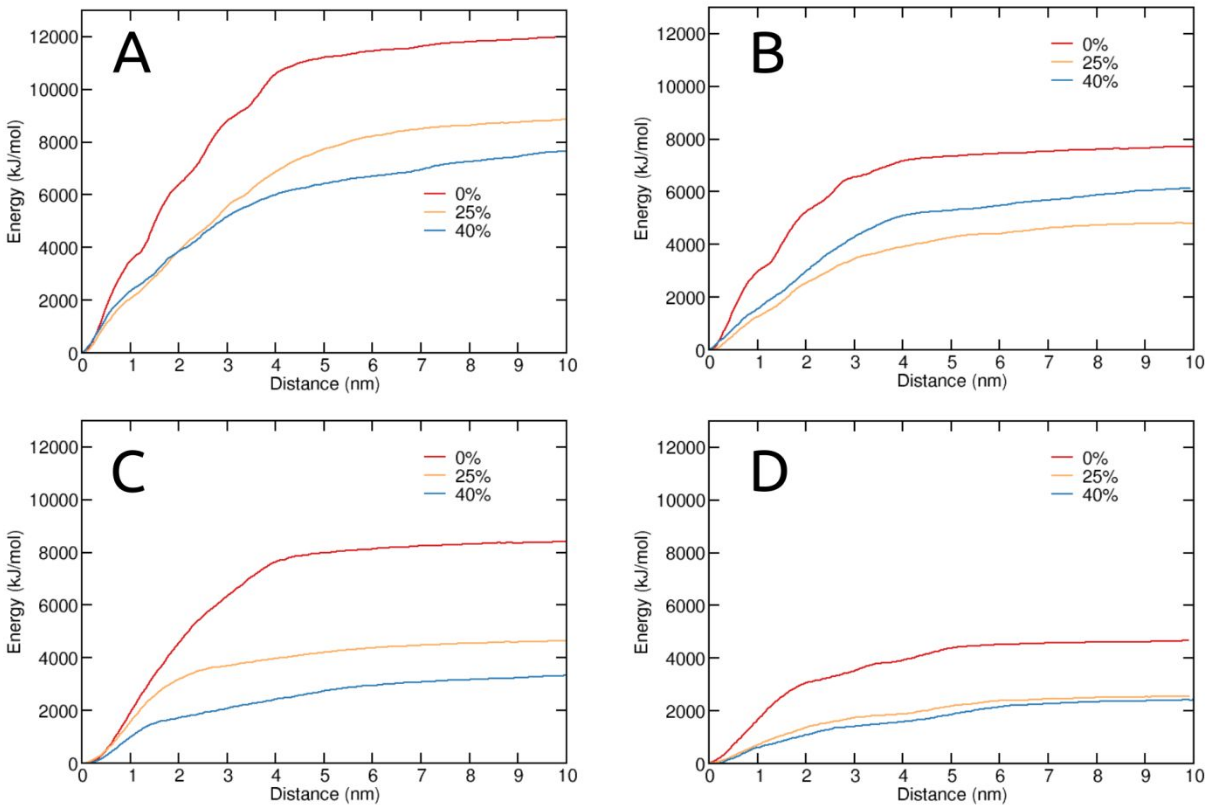

Figure S7 Interaction energy (calculated from integration of the force-dissplacement curves) for systems A) Ax_3@25; B) Ax_3@100; C) Tr_3@25; D) Tr_3@100.
